# CAF-Driven EMT and ECM Remodeling Programs Promote Mesothelioma Progression

**DOI:** 10.3390/cancers18152424

**Published:** 2026-07-28

**Authors:** Licun Wu, Hana Yun, Hamed Yasavoli Sharahi, Fatemeh Zaeimi, Marc de Perrot

**Affiliations:** 1 Latner Thoracic Surgery Research Laboratories, Division of Thoracic Surgery, Toronto General Hospital, University Health Network, University of Toronto, Toronto, ON M5G 1L7, Canada; licun.wu1@uhn.ca (L.W.); zhihong_yun@yahoo.ca (H.Y.); hamed.yasavolisharahi@uhn.ca (H.Y.S.); fatemeh.zaeimi@uhn.ca (F.Z.); 2 Princess Margaret Cancer Centre, University Health Network, Toronto, ON M5G 1L7, Canada; 3 Department of Immunology, University of Toronto, Toronto, ON M5S 1A8, Canada

**Keywords:** mesothelioma, cancer-associated fibroblasts (CAF), epithelial–mesenchymal transition (EMT), extracellular matrix (ECM) remodeling, tumor microenvironment (TME), mechanotransduction, stromal–tumor interactions

## Abstract

Background: The aggressive progression of mesothelioma is driven not only by intrinsic tumor cell plasticity but also by dynamic interactions between tumor cells and the surrounding stromal microenvironment; however, the mechanisms by which stromal populations regulate epithelial–mesenchymal transition (EMT), tumor evolution, and therapeutic resistance remain unclear. Methods: We integrated longitudinal transcriptomic profiling with single-cell RNA sequencing in a murine intraperitoneal mesothelioma model spanning disease progression from week 0 to week 8 to identify stromal-EMT programs associated with tumor progression. In vitro fibroblast–tumor co-culture systems were used to assess how different fibroblast-to-tumor cell ratios influence transcriptional reprogramming and mesenchymal transition. Results: Single-cell analysis revealed marked stromal heterogeneity, identifying eight distinct cancer-associated fibroblast (CAF) subtypes: myCAF, mCAF, iCAF, TGF-βCAF, vCAF, plCAF, apCAF, and meCAF. These subsets showed specialized transcriptional programs associated with developmental signaling and metabolic adaptation. Functional analyses demonstrated coordinated intercellular communication across six interconnected modules, including mesenchymal transition, fibrotic remodeling, TGF-β/metabolic adaptation, adhesion signaling, WNT activation, and inflammatory crosstalk. Notably, mCAF, TGF-βCAF, and plCAF populations showed transcriptional convergence, suggesting cooperative formation of a desmoplastic, immunoregulatory niche. Co-culture experiments confirmed that fibroblast-derived signaling induces ratio-dependent transcriptional changes, promoting a shift from epithelioid to mesenchymal phenotypes. Conclusions: These findings highlight stromal regulation of EMT as a key driver of mesothelioma progression, sarcomatoid transition, and therapy resistance, and identify tumor–stroma signaling networks as potential therapeutic targets.

## Simple Summary

This study shows that mesothelioma grows aggressively by leveraging a conversational network between cancer cells and their surrounding neighborhood. By tracking the disease over an eight-week span and analyzing individual cells, we identified eight distinct types of Cancer-Associated Fibroblasts (CAFs), each performing specialized duties like building protective physical barriers, feeding the tumor via new blood vessels, or hiding it from the immune system. Notably, three of these helper populations join forces to construct a dense, defensive wall around the tumor. Lab experiments confirmed that signals from these fibroblasts force less aggressive cancer cells to alter their metabolism and shape-shift into a highly dangerous, treatment-resistant state. By mapping out this detailed communication blueprint, the study successfully exposes the microenvironmental evolution and highlights these cellular conversations as prime targets for developing new therapies to halt tumor progression.

## 1. Introduction

Malignant mesothelioma is an aggressive cancer arising from the mesothelial lining, most often the pleura, and is strongly linked to asbestos exposure after a latency period of 30–50 years [1]. Even with modern treatment, the prognosis remains poor, with median overall survival typically ranging from 12 to 18 months [2]. Several factors drive this unfavorable outcome. The tumor grows in a diffuse, locally invasive manner that makes complete surgical resection difficult and fuels early recurrence. It also shows marked resistance to conventional chemotherapy, and while immunotherapy has helped some patients, durable responses remain limited to a minority [3]. Most people are diagnosed at an advanced stage, which narrows treatment options and leaves current approaches largely palliative [1].

The behavior of mesothelioma is shaped not only by malignant cells but also by the surrounding tumor microenvironment (TME), a complex mix of cancer-associated fibroblasts (CAF), endothelial cells, and immune populations that together support tumor growth and persistence. Bidirectional interactions between mesothelioma cells and the stroma drive disease progression. Stromal signaling remodels the extracellular matrix, promotes invasion, and suppresses immune activity, impairing cytotoxic T cell function and fueling resistance to chemotherapy and immunotherapy [4,5]. Fibroblast-driven remodeling, in particular, is a key contributor to aggressiveness and treatment failure. The composition of the TME varies across mesothelioma subtypes and genomic contexts, pointing to stromal programs as integral drivers of disease biology [1].

Within this TME, CAFs are abundant and actively shape tumor behavior far beyond being passive structural components [6]. They are associated with progression through two main mechanisms: remodeling the extracellular matrix (ECM) and suppressing anti-tumor immunity. CAFs produce and organize ECM proteins like collagens and fibronectin, altering tissue stiffness and architecture to promote invasive growth and therapy resistance [7]. At the same time, they secrete cytokines and chemokines that limit cytotoxic T cell infiltration and foster an immunosuppressive niche [8]. Together, these activities help establish a stromal environment that supports tumor invasion, survival, and malignant progression.

Epithelial–mesenchymal transition (EMT) is a biological process through which epithelial cells lose adhesion and gain invasive, motile traits, a key mechanism driving tumor plasticity and adaptation to pressures like hypoxia and therapy [9]. In mesothelioma, activation of EMT programs enables cells to detach, remodel the ECM, and invade locally [1]. These programs have been linked to matrix remodeling, mechanotransduction, and treatment resistance, reflecting their functional importance beyond a simple epithelial-to-mesenchymal shift [2]. EMT is now understood as a context-dependent, reversible process shaped by continuous crosstalk between tumor cells and the surrounding stroma [9].

In this study, we examine stromal EMT programs and their association with distinct CAF ecosystems, extracellular matrix remodeling and immune adaptation. By integrating transcriptional, stromal and immune profiling, we aim to characterize stromal states, moving beyond tumor cell-centric models to define EMT as a context-dependent program that coordinates tumor-stroma interactions and disease progression. Understanding these genotype-dependent stromal architectures may help explain biological heterogeneity in mesothelioma and reveal new opportunities for targeted and combinatorial therapies.

## 2. Materials and Methods

### 2.1. Cell Lines and Mice

Cells: Murine mesothelioma RN5 cell line, a biphasic line derived from Nf2 heterozygous C57BL/6 mice, was established in our team in collaboration with the University of Zurich and University of Freiburg, Switzerland, and maintained in RPMI 1640 supplemented with 10% fetal bovine serum and 1% penicillin/streptomycin (Gibco^TM^, Thermo Fisher Scientific, Burlington, ON, Canada). Tumor cells (2 × 10^6^/200 µL PBS/mouse) were injected intraperitoneally into 6–8-week-old wild-type mice [10]. Murine fibroblast cell line CCL-206 was provided by ATCC (Manassas, VA, USA). This is a normal fibroblast cell line isolated from the lung tissue of healthy ddY strain mice (https://www.atcc.org/products/ccl-206) (Accessed on 15 May 2025). Both cell lines had been confirmed mycoplasma-free.

Mice: Female C57BL/6 mice (6–8 weeks old) were obtained from The Jackson Laboatory (Bar Harbor, ME, USA). All animal experiments were conducted in compliance with the Animal Use Protocol approved by the University Health Network (AUP #3399.28).

### 2.2. Experimental Design and Dataset Sources

**Figure 1:**
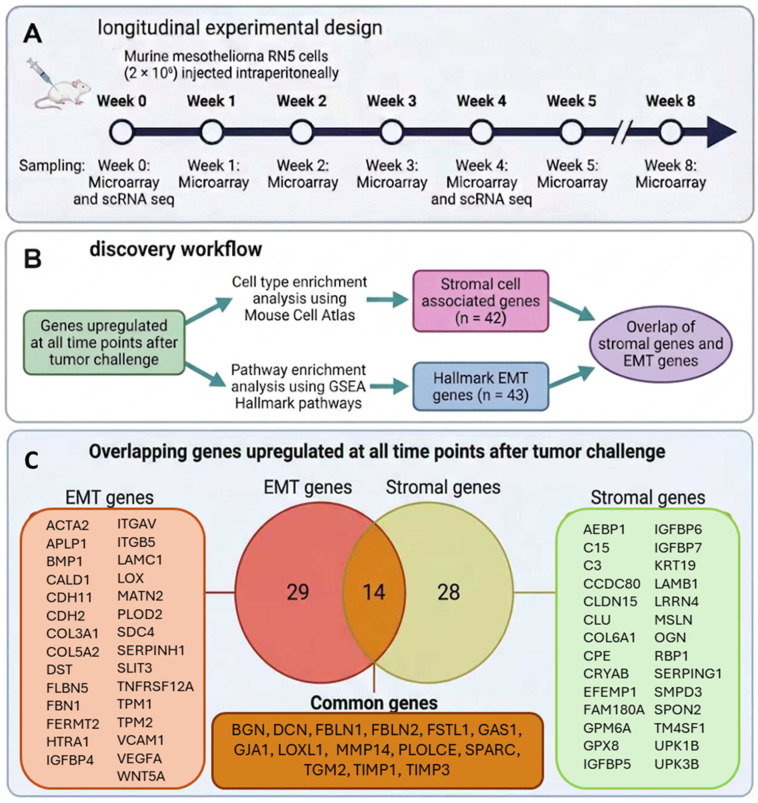
Study design, stromal and EMT gene signatures identified in a murine intraperitoneal model. (**A**) Murine mesothelioma RN5 cells (2 × 10^6^) were injected intraperitoneally, and tumors were profiled longitudinally from week 0 to week 8 using microarray, with single-cell RNA sequencing performed at weeks 0 and 4. (**B**) Genes consistently upregulated across all time points after tumor challenge were analyzed for cell-type enrichment using the Mouse Cell Atlas and for pathway enrichment using Gene Set Enrichment Analysis (GSEA). (**C**) Stromal cell-associated genes (*n* = 42; WebGestalt 2024) and Hallmark epithelial–mesenchymal transition (EMT) genes (*n* = 43; MSigDB) were compared. Venn diagram analysis identified 14 genes shared between stromal and EMT signatures, indicating a convergent stromal-EMT transcriptional program during tumor progression. Gene lists for EMT, stromal, and overlapping signatures are shown.

The experimental design is illustrated in Figure 1A. Forty mice were included in this experiment. Five naïve mice were used as controls, while 35 tumor-bearing mice were sacrificed at weekly time points, 5 mice per group. After intraperitoneal injection of tumor cells, peritoneal lavage fluid was collected at multiple time points to profile gene expression within the tumor microenvironment (TME), with naïve mice and cultured tumor cells serving as controls.

Total RNA from lavages was used for microarray analysis, while fresh single cells were processed for single-cell RNA sequencing (scRNA-seq) following standard protocols. Detailed descriptions of the transcriptomic and scRNA-seq workflows include RNA extraction, platforms, sequencing parameters, and data processing steps in our previous study [10]. Single-cell data were analyzed using Loupe Cell Browser v7 (10× Genomics). A reference mouse genome (mm10) was used to filter out cells with high mitochondrial UMIs, indicative of poor quality or dying cells. Globally distinguishing genes were carried forward for downstream analyses [10].

### 2.3. Co-Culture of Tumor Cells and Fibroblasts

Direct contact monolayer model: Tumor cells-murine mesothelioma cell line RN5 and mouse lung fibroblast cell line CCL-206 were grown together in a 6-well plate with RPMI1640 medium and 10% FBS. Culture conditions for CCL-206 are similar to RN5 cells: RPMI1640 medium and 10% FBS. The passage numbers used in the experiments were 3–5 passages. This method allows for physical interaction and direct cell–cell signaling. The ratios of tumor cells (T) to fibroblasts (F) were optimized to ensure physiological relevance: T:F = 0:1 (0:50,000), fibroblasts alone; T:F = 1:1 (25,000:25,000); T:F = 1:5 (10,000:50,000); T:F = 5:1 (50,000:10,000). On day 3 of culture, cells were harvested to run scRNAseq. Cell viability was assessed prior to library preparation and exceeded 95%. The 3-day culture duration was selected to capture early dynamic interactions between tumor and stromal cells while minimizing overgrowth and secondary adaptation effects. This time frame provides a balance between physiological relevance and experimental reproducibility.

### 2.4. Differential Expression Analysis

Differential gene expression analysis was performed using normalized transcriptomic datasets to identify genes significantly altered across experimental conditions and longitudinal time points. Murine scRNA-seq data were processed using Loupe Cell Browser (10× Genomics, v6.x) for initial visualization and quality assessment, and further analyzed in Seurat (v4.x, R v4.x). Raw counts were normalized using the LogNormalize method, with mitochondrial gene content used as an additional quality-control metric. For scRNA-seq, differential expression analysis was conducted using Seurat-based statistical methods appropriate for sparse single-cell data, incorporating cell-level normalization and covariate adjustment. Genes expressed in fewer than three cells were excluded. Differentially expressed genes were defined based on an absolute fold change > 2.0 and adjusted *p* < 0.05, unless otherwise specified [11].

### 2.5. Pathway Enrichment Analysis and Gene Set Curation of Mesothelioma-Associated Gene Signatures

Genes that were consistently upregulated across all post-tumor challenge time points were identified by intersecting differentially expressed gene lists derived at each time point using predefined significance thresholds (e.g., adjusted *p* < 0.05 and fold-change cutoff). The resulting gene set was subjected to cell-type enrichment analysis using the Mouse Cell Atlas to infer predominant cellular origins. Functional pathway enrichment was performed using Gene Set Enrichment Analysis (GSEA) with standard parameters and curated gene sets. Three gene signatures associated with mesothelioma biology were analyzed for pathway enrichment: an EMT-related 29-gene set (EMT29), a stromal-derived 28-gene set (Stromal28), and a 14-gene common signature (Common14).

### 2.6. Gene Ontology Enrichment Analysis

Gene Ontology (GO) enrichment was performed to identify overrepresented biological processes (BP), molecular functions (MF), and cellular components (CC) in differentially expressed gene sets. Overrepresentation analysis (ORA) was conducted using a hypergeometric test against a background of all detected genes, with FDR correction for multiple testing. GO terms with an adjusted *p* < 0.05 were considered significantly enriched and ranked by enrichment score or gene ratio [11]. Hallmark pathways and Gene Ontology (GO) enrichment of EMT29, Stromal28, and Common14 gene sets were performed using the software and database versions used (e.g., WebGestalt, current release), the definition of the background gene universe (all expressed genes passing filtering criteria), and the statistical framework applied. Enrichment significance was calculated using over-representation analysis, with multiple-testing correction (FDR), and results were ranked based on adjusted *p*-values and enrichment ratios.

### 2.7. EMT Gene Signature Scoring

EMT activity was quantified using a curated gene signature derived from the Hallmark EMT gene set (MSigDB, Hallmark Epithelial–Mesenchymal Transition; https://www.gsea-msigdb.org/gsea/msigdb) (Accessed on 10 January 2025), distributed under the Creative Commons Attribution 4.0 International License.

For each sample or cell population, EMT scores were calculated by aggregating the normalized expression (e.g., mean of z-normalized values) of signature genes after gene-wise scaling, permitting comparisons of EMT program activation across experimental groups or time points [12]. Cell type annotation and over-representation analysis were performed using the WebGestalt toolkit (WEB-based GEne SeT AnaLysis Toolkit). Gene expression profiles were mapped to reference datasets, including the Mouse Cell Atlas, enabling the inference of cell-type identities based on enriched gene signatures.

### 2.8. Sankey and Dot Plot Visualization

To visualize gene–pathway relationships and enriched gene programs, Sankey plots were generated to map individual genes to significantly enriched Hallmark pathways, depicting connectivity and relative contributions of each gene. Sankey dot plots were generated using the online tool SRplot 2025 (https://www.bioinformatics.com.cn/srplot) (Accessed on 10 January 2025), a web-based platform designed for visualizing GO and pathway enrichment results.

Dot plots were used to summarize enrichment results, with the x-axis representing gene ratios, dot size indicating the number of contributing genes, and color denoting enrichment significance (−log_10_ *p*-value) [13].

### 2.9. CAF Subtype Identification and Mapping

Using AI-assisted analytical frameworks, we have described the computational approach for CAF subtype assignment, including the underlying algorithms and reference datasets applied. CAF subtypes, including myofibroblastic (myCAFs), inflammatory (iCAFs), matrix CAFs, and antigen-presenting (apCAFs), were annotated using a reference framework from published scRNA-seq studies [14], then refined with mesothelioma-specific data to identify populations such as vascular-associated and TGFβ-responsive CAFs. We further detailed the quantitative assessment of similarity between EMT/stromal gene signatures and CAF subtypes using scoring and correlation-based methods. Marker genes were grouped into functional categories (e.g., contractile, inflammatory, ECM regulatory) to enable comparative characterization, revealing that the EMT29 program aligns with a myCAF-like phenotype due to enrichment of contractile and matrix remodeling markers (*ACTA2*, *TPM1/2*, *COL3A1*), while the Stromal28 program matches an iCAF-like profile through immune regulatory factors (*C3*, *C1S*, *IGFBP5-7*) [14]. Additional subtypes (TGFβ-CAF, vCAF, pleuralCAF, meCAF) were annotated based on established markers and prior studies on CAF heterogeneity [15,16].

### 2.10. Identification of Adaptation Receptor-Ligand Axes

The analysis was based on curated ligand–receptor interaction databases integrated with gene expression data, rather than de novo probabilistic modeling. To explore stromal communication in the mesothelioma tumor microenvironment, genes were classified as Adaptation Receptors (AdR)-cell-surface or signaling receptors or Adaptation Ligands (AdL)-secreted ECM proteins, cytokines, and regulators of growth factor availability, using Gene Ontology, UniProt, and matrisome annotations [17].

Receptor–ligand pairs were mapped using a cell-cell communication framework that integrates curated ligand–receptor interactions with single-cell transcriptomic expression, retaining only experimentally supported interactions [18]. These pairs were assembled into AdR-AdL networks to identify communication axes, emphasizing ECM-derived ligands engaging tumor or fibroblast receptors that may drive cellular adaptation in the pleural tumor microenvironment [18].

### 2.11. Statistical Analysis

Differential gene expression was analyzed using normalized scRNA-seq data. Genes were considered significant if they passed moderated *t*-tests with Benjamini–Hochberg FDR correction (adjusted *p* < 0.05) [11]. Single-cell RNA-seq data were analyzed using Loupe Cell Browser (v7, 10× Genomics) for visualization and exploratory analysis. Only genes expressed in a minimum fraction of cells and meeting predefined thresholds (log2FC > 1, adjusted *p* < 0.05) were retained for downstream analyses. Model-based methods for sparse single-cell counts were applied, including cell-level normalization and covariate adjustment. Functional analyses included GO enrichment and EMT scoring to measure mesenchymal program activity [12], while CAF subtypes and AdR-AdL interactions were mapped using single-cell frameworks and curated ligand–receptor databases [14,18].

## 3. Results

### 3.1. Longitudinal Profiling Identifies a Convergent Stromal-EMT Transcriptional Program During Mesothelioma Progression

Using a murine intraperitoneal mesothelioma model, we performed longitudinal transcriptional profiling from tumor implantation to established disease. Bulk microarray analysis (weeks 0–8), complemented by single-cell RNA sequencing at weeks 0 and 4, identified sustained induction of a core gene set following tumor challenge. Stromal-associated genes (*n* = 42; WebGestalt) and EMT genes (*n* = 43; Hallmark, MSigDB) were compared to assess overlap and shared programs (Figure 1A,B).

Cell-type enrichment showed that persistently upregulated genes were primarily associated with stromal and mesenchymal compartments. Comparison of stromal and EMT gene sets revealed a subset of overlapping transcripts, defining a shared stromal-EMT signature that emerged early and remained stable during tumor progression (Figure 1C). This early and sustained overlap indicates coordinated activation of extracellular matrix remodeling and EMT programs, suggesting tight coupling between stromal reprogramming and tumor cell plasticity from the onset of disease.

### 3.2. Functional Partitioning of EMT, Inflammatory Stroma, and Matrix Cores in Mesothelioma

**Figure 2:**
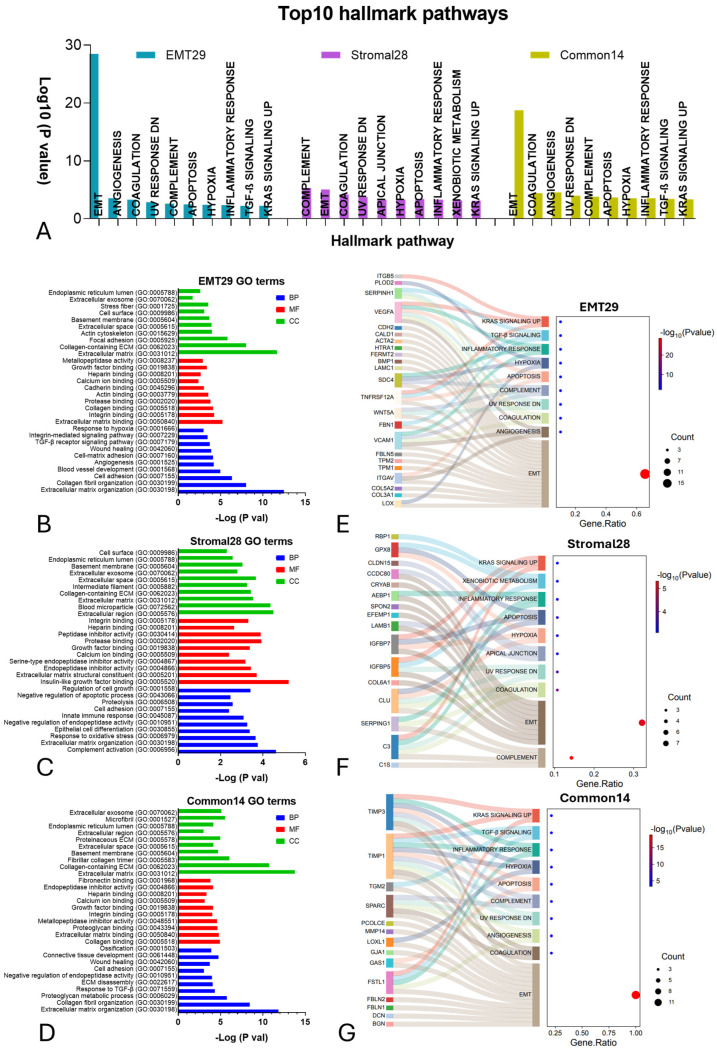
Hallmark pathways and Gene Ontology enrichment of EMT29, Stromal28, and Common14 gene sets. (**A**) Top 10 enriched Hallmark pathways for EMT29, Stromal28, and Common14 gene sets shown as −log(*p* value). (**B**–**D**) Gene Ontology (GO) enrichment analysis for EMT29 (**B**), Stromal28 (**C**), and Common14 (**D**), highlighting significantly enriched biological process (BP), molecular function (MF), and cellular component (CC) terms. EMT29 is enriched for ECM organization, adhesion, and mechanotransduction processes; Stromal28 shows stromal ECM remodeling and inflammatory responses; Common14 captures shared ECM structural and regulatory functions linking EMT and stromal programs. (**E**–**G**) Hallmark pathway enrichment and gene-pathway associations. Sankey plots (**left**) show mapping of input genes to enriched Hallmark pathways. Dot plots (**right**) summarize pathway enrichment by gene ratio (x-axis), with dot size indicating gene count and color representing significance (−log10 *p*-value). EMT exhibits the highest enrichment, followed by angiogenesis, coagulation, and inflammatory signaling pathways in EMT29 gene set (**E**) compared with others (**F**,**G**). Pathway nodes (**middle**) are shown in distinct colors to differentiate enriched biological pathways, and the connecting ribbons inherit the color of their corresponding pathway to facilitate visualization of gene-pathway relationships.

Hallmark Pathway analysis delineates a stratified functional landscape among the three transcriptional programs. EMT29 is overwhelmingly dominated by the EMT hallmark (−log10 *p*  >  25), accompanied by strong enrichment for angiogenesis and inflammatory signaling. Common14 functions as a conserved secondary EMT core, whereas Stromal28 displays selective enrichment for complement and inflammatory responses, consistent with specialization toward a fibrotic stromal niche (Figure 2A). Gene Ontology analysis further resolves a functional hierarchy during mesothelioma progression. EMT29 is enriched for mechanotransduction and cell-matrix adhesion terms, including extracellular matrix organization and integrin binding, reflecting acquisition of invasive mesenchymal phenotypes. Stromal28 is preferentially associated with fibrotic remodeling and inflammation, with enrichment for complement activation and IGF binding, suggesting engineering of a growth-factor-sequestering, immune-excluded niche. Common14 captures a conserved ECM program, enriched for collagen fibril organization and protease inhibition, defining baseline structural stabilization and metabolic coupling required for tumor persistence. Together, these data support a coordinated model in which Common14 establishes structural integrity, Stromal28 shapes a protective inflammatory microenvironment, and EMT29 drives mechanically coupled invasion, generating a fortified tumor ecosystem associated with malignancy and therapeutic resistance (Figure 2B–D).

Sankey dot plots provide high-resolution mapping of gene-to-pathway contributions underlying functional divergence. EMT29 acts as a mechanotransduction engine, with LOX, COL3A1, COL5A2, ITGAV, and VCAM1 contributing disproportionately to the EMT hallmark. Secondary flows toward angiogenesis and hypoxia indicate concurrent remodeling of the metabolic landscape. Stromal28 defines an immune-excluded niche, with dominant contributions from C3, C1S, and SERPING1 to complement signaling, alongside IGFBP5, IGFBP7, and AEBP1 linking inflammatory and KRAS-associated pathways. Common14 serves as a conserved matrix core, with FBLN1/2, BGN, and DCN supporting EMT-associated structure, while TIMP1/3 and MMP14 balance coagulation and inflammatory signaling. Bridging genes such as GJA1 and TGM2 link ECM organization to cellular coupling (Figure 2E–G). Thus, while all programs converge on EMT, EMT29 operates through mechanical tension, Stromal28 through inflammatory niche engineering, and Common14 through conserved matrix architecture.

### 3.3. Distinct CAF Subtypes Orchestrate Structural, Inflammatory and Metabolic Niches in Mesothelioma

Mapping these transcriptomic signatures across the cellular landscape reveals a coordinated division of labor among CAF subtypes (Table 1). Matrix-producing CAFs (matrixCAF, mCAF) and TGFβ-activated CAFs (TGFβCAF) emerge as the principal structural architects of the mesothelioma microenvironment, exhibiting strong enrichment across all three transcriptional programs. MatrixCAFs, in particular, express a comprehensive repertoire of collagen-modifying enzymes, including LOX and PLOD2, together with core basement membrane components such as FBLN1, FBLN2, and FBLN5, consistent with a critical role in ECM assembly and stabilization.

Distinct CAF subpopulations execute more specialized niche-building functions. Pleural-derived CAFs (pleuralCAF, plCAF) are uniquely defined by the Stromal28 program and express site-specific anchoring markers, including MSLN and CLDN15, identifying them as key contributors to mesothelial-associated fibrosis. Vascular CAFs (vCAF) preferentially engage pro-angiogenic and mechanosensory pathways through VEGFA and integrin signaling (*ITGAV/ITGB5*), linking matrix remodeling to vascular adaptation.

Inflammatory and metabolic functions are similarly compartmentalized. Inflammatory CAFs (iCAF) concentrate complement pathway activation (*C3*, *C1S*) and growth-factor sequestration (*IGFBP5*, *IGFBP6*, *IGFBP7*), whereas metabolic CAFs (meCAF) are enriched for lipid handling and antioxidant transport programs, including RBP1 and GPX8. In contrast, antigen-presenting CAFs (apCAF) show no significant enrichment across EMT29, Stromal28, or Common14, indicating functional specialization distinct from core mesenchymal and structural remodeling programs.

**Figure 3:**
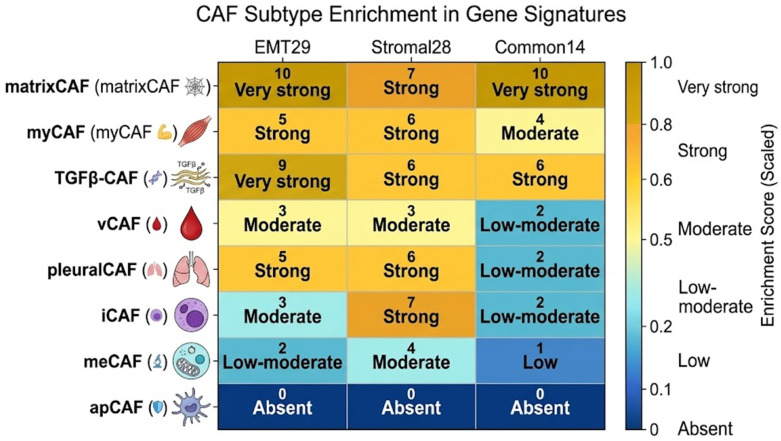
Enrichment of Cancer-Associated Fibroblast (CAF) subtypes across established gene signatures. Heatmap representing the enrichment scores of eight distinct CAF subtypes across three validated gene sets: EMT29, Stromal28, and Common14. The subtype order is displayed as matrixCAF, myCAF, TGFβ-CAF, vCAF, pleuralCAF, iCAF, meCAF, and apCAF. Data are presented as scaled enrichment scores, with color intensity reflecting the degree of gene representation ranging from Absent (dark blue) to Very strong (gold). Numerical values and categorical descriptors within each cell denote the absolute gene counts and the corresponding enrichment strength (e.g., matrixCAF shows a “Very strong” representation with 10 genes in the EMT29 set). Biological icons adjacent to the y-axis labels illustrate the functional characteristics of each subtype, such as ECM fibers for matrixCAF and contractile muscle fibers for myCAF. Enrichment strength is scaled from 0 to 1.0 based on the maximum gene representation observed.

Consistent with these observations, enrichment heatmaps identify a clear functional hierarchy among CAF subtypes in mesothelioma. MatrixCAFs and TGFβ-CAFs act as dominant drivers of tumor remodeling, with very strong to strong enrichment across EMT29 and Common14. PleuralCAFs display highly specific enrichment for Stromal28, defining them as the principal architects of the mesothelial fibrotic niche. Other CAF populations, including iCAFs and meCAFs, show limited engagement with these structural programs, while apCAFs are entirely absent, underscoring a clear division between matrix-remodeling and secondary support functions (Figure 3).

### 3.4. AdR-AdL Signaling Programs Coordinate EMT and Stromal Niche Remodeling in Mesothelioma

**Figure 4:**
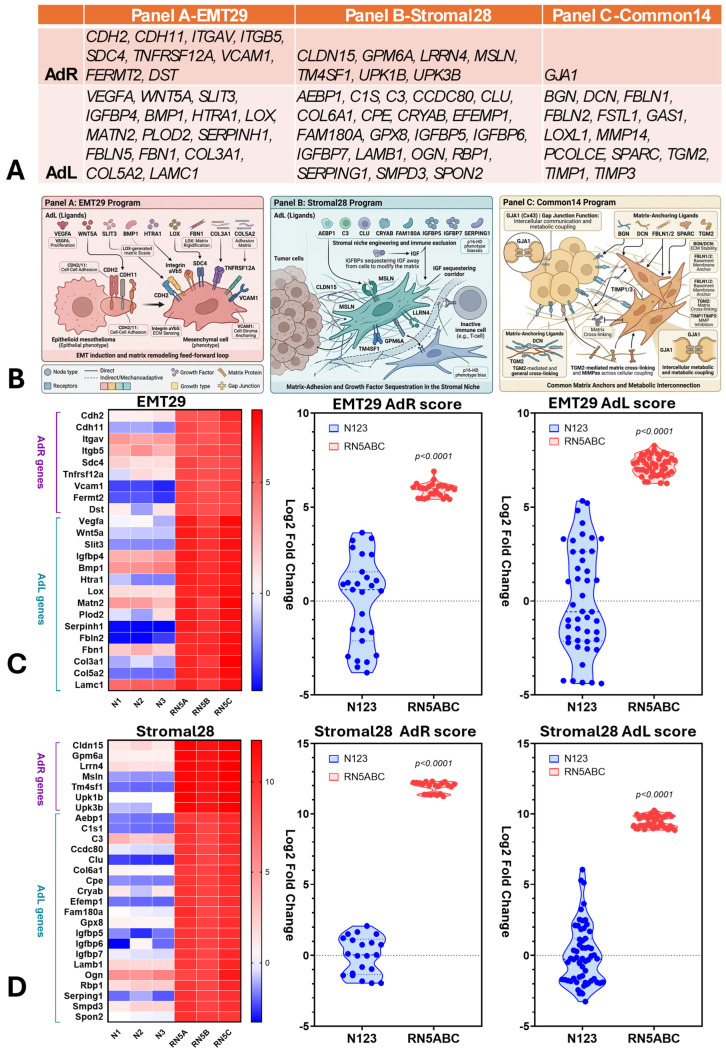
AdR-AdL Signaling Axes Across Mesothelioma Cellular Programs. (**A**) The table shows adaptation receptors and ligands (AdR-AdL) from each gene set. (**B**) Panel A: EMT29 Program (Epithelial-to-Mesenchymal Transition): Illustrating the transition from an epithelioid to a mesenchymal phenotype. Key drivers include cadherin switching (*CDH2/11*), ECM sensing via Integrin αVβ5, and cell-stroma anchoring mediated by VCAM1. Growth factors (*VEGFA*, *WNT5A*) and matrix modifiers (*LOX*, *FBN1*) create a feed-forward loop promoting invasion. Panel B: Stromal28 Program (Niche Engineering): Details the construction of the stromal microenvironment. MSLN (Mesothelin) and CLDN15 anchor tumor cells to the matrix, while IGFBP5/7 sequester IGF to modulate the “IGF-sequestering corridor.” This program is biased toward the p16-HD phenotype, facilitating immune exclusion. Panel C: Common14 Program (Matrix Anchors and Metabolic Coupling): Showing the foundational structural and communicative network shared across subtypes. GJA1 (Connexin 43) mediates intercellular metabolic coupling, while a suite of matrix proteins (*BGN*, *DCN*, *FBLN1/2*) and cross-linkers (*TGM2*) provide mechanical stability. TIMP1/3 regulate the remodeling process by inhibiting matrix metalloproteinases (MMPs). Visual Key: Solid lines indicate direct signaling/binding; dashed lines represent indirect mechanoadaptive effects. (**C**) Validation of the EMT29 Program. Heatmap (**left**) illustrates a sharp contrast in AdR and AdL gene expression between groups of naïve and RN5-nearing mice. Accompanying violin plots (**right**) show a statistically significant elevation (*p* < 0.0001) in both EMT29 AdR and AdL scores for the RN5ABC group, confirming active EMT. (**D**) Validation of the Stromal28 Program. Heatmap (**left**) shows broad upregulation of stromal engineering genes in RN5ABC cohort. Quantitative analysis via violin plots (**right**) demonstrates a highly significant increase (*p* < 0.0001) in Stromal28 AdR and AdL scores compared with N123, indicating advanced matrix remodeling and niche construction in these samples.

To define signaling networks underlying mesothelioma progression, we mapped AdR-AdL interactions across the EMT29, Stromal28, and Common14 programs (Figure 4A). EMT29 defined a mechanoadaptive mesenchymal program enriched for cadherin switching (*CDH2*, *CDH11*), integrin-mediated ECM sensing (ITGAV/ITGB5), and VCAM1-dependent cell–stroma interactions (Figure 4B, panel A). Concurrent induction of *LOX*, *FBN1*, *VEGFA*, and *WNT5A* supported matrix remodeling, niche stiffening, and invasive EMT-associated signaling.

Stromal28 represented a stromal niche engineering program characterized by *MSLN*, *CLDN15*, and IGF-sequestering factors (*IGFBP5*, *IGFBP7*), consistent with matrix organization and immune exclusion (Figure 4B, panel B). This program was preferentially enriched in p16 homozygous deletion-associated tumors. In parallel, Common14 defined a conserved structural network involving *GJA1*-mediated metabolic coupling and coordinated ECM remodeling through *BGN*, *DCN*, *FBLN1/2*, *TGM2*, *TIMP1/3*, and *MMP14* (Figure 4B, panel C).

Validation analyses demonstrated marked activation of EMT29 and Stromal28 programs in tumor-bearing RN5 mice compared with naïve controls (Figure 4C,D). Heatmaps revealed broad induction of AdR and AdL genes, while composite EMT29 and Stromal28 AdR/AdL scores were significantly increased in RN5ABC samples (*p* < 0.0001), consistent with activation of EMT, stromal remodeling, and niche specialization during mesothelioma progression. These interactions were interpreted as putative and hypothesis-generating rather than mechanistically confirmed. Therefore, experimental validation will be required to substantiate these signaling relationships.

### 3.5. Fibroblast Abundance Reshapes Mesothelioma Transcriptional States Through Tumor–Stroma Interactions

**Figure 5:**
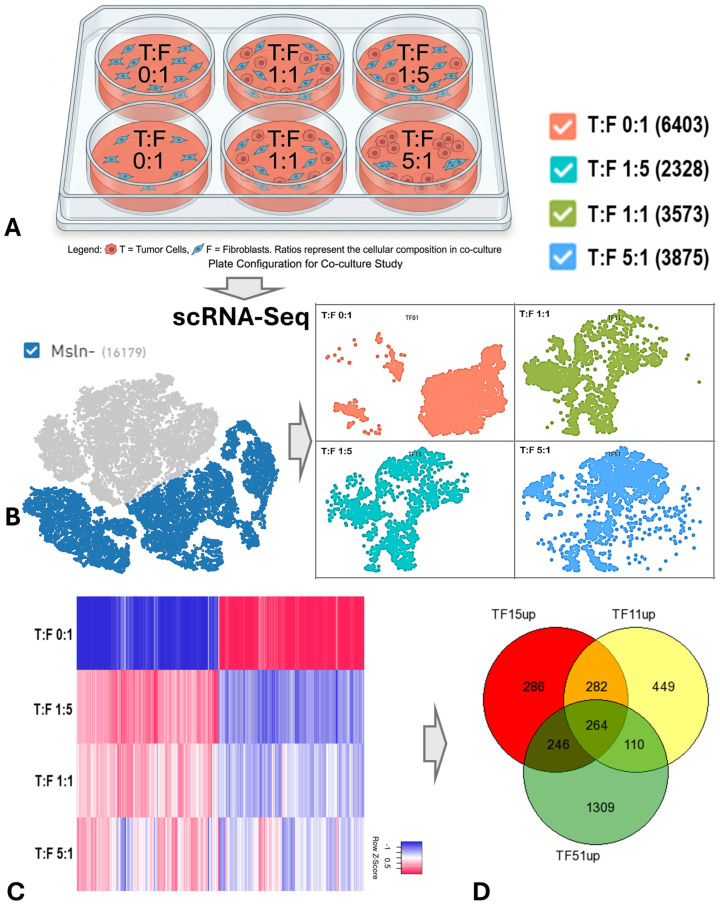
Tumor fibroblast co-culture mimicking tumor microenvironment. (**A**) Schematic of the experimental design showing tumor cells co-cultured with fibroblasts at indicated tumor:fibroblast (T:F) ratios (0:1, 1:5, 1:1, 5:1). T:F = 0:1 (0:50,000), fibroblast alone; T:F = 1:1 (25,000:25,000); T:F = 1:5 (10,000:50,000); T:F = 5:1 (50,000:10,000). T: tumor cells-murine mesothelioma cell line RN5; F: fibroblast cell line CCL-206. On day 3 of culture, cells were harvested to run scRNAseq. (**B**) Single-cell transcriptomes were projected by tSNE, with Msln-negative cells highlighted and cells stratified by T:F condition. Condition-specific tSNEs illustrate distinct cellular distributions across ratios, with total cell numbers indicated. Blue color highlighted Msln− cell population for further analysis. (**C**) A heatmap of differentially expressed genes (row z-score) summarizes ratio-dependent transcriptional programs. (**D**) Venn diagram showing the overlap of upregulated genes among T:F conditions, highlighting shared and unique responses to fibroblast abundance.

To model stromal regulation of mesothelioma progression, RN5 mesothelioma cells were co-cultured with CCL-206 fibroblasts across varying tumor:fibroblast (T:F) ratios followed by single-cell RNA sequencing analysis (Figure 5A). tSNE projection revealed distinct cellular architectures across co-culture conditions, indicating that fibroblast abundance dynamically alters tumor cell composition and transcriptional state (Figure 5B). Msln-negative populations showed marked redistribution under fibroblast-enriched conditions, consistent with stromal-driven cellular reprogramming.

Differential expression analysis identified ratio-dependent transcriptional programs associated with extracellular matrix remodeling, inflammatory signaling, stress adaptation, and mesenchymal transition (Figure 5C). Overlap analysis further demonstrated both conserved and condition-specific gene signatures across T:F ratios, indicating that fibroblast abundance quantitatively modulates discrete stromal and EMT-associated programs during tumor progression (Figure 5D).

### 3.6. Distinct CAF Subtypes Exhibit Specialized Functional Programs in Mesothelioma

**Figure 6:**
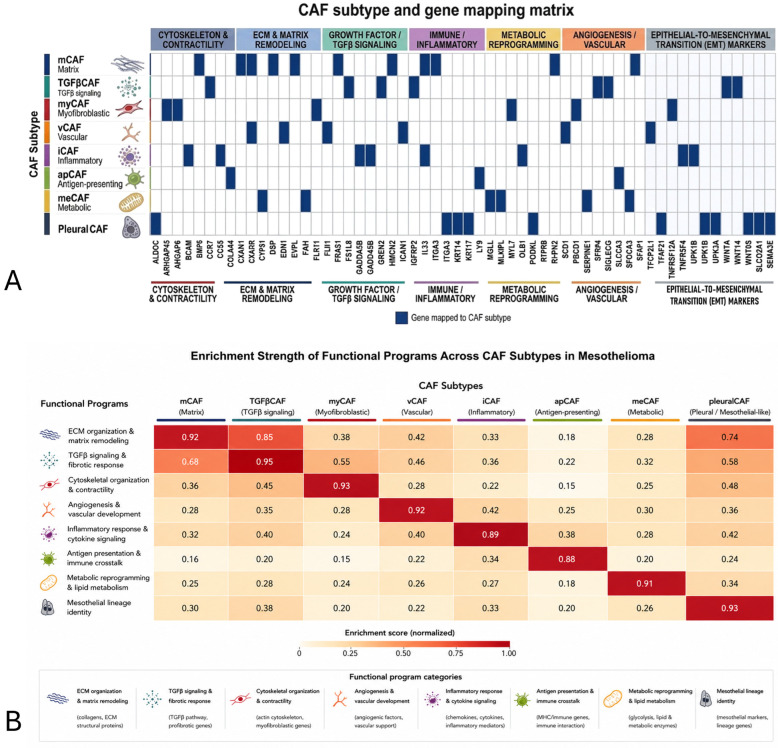
Functional Characterization and Enrichment of CAF Subtypes in Mesothelioma. (**A**) CAF subtype gene mapping matrix. Heatmap displaying the association of distinct Cancer-Associated Fibroblast (CAF) subtypes (y-axis) with specific functional gene signatures (x-axis). Functional categories include Cytoskeleton & Contractility, ECM & Matrix Remodeling, Growth Factor/TGFβ Signaling, Immune/Inflammatory, Metabolic Reprogramming, Angiogenesis/Vascular, and Epithelial-to-Mesenchymal Transition (EMT) Markers. Blue squares indicate a validated gene mapping to the respective CAF subtype, highlighting the unique molecular fingerprints of populations like mCAF (matrix-rich) and apCAF (antigen-presenting). (**B**) Enrichment strength of functional programs. Normalized heatmap quantifying the dominance of specific biological programs across CAF subtypes. High diagonal scores (≥0.88) confirm the functional specialization of each subtype, such as vCAF in angiogenesis and meCAF in metabolic reprogramming. Moderate overlap between mCAF, TGF-βCAF, and pleuralCAF in ECM-related programs suggests a synergistic contribution to the desmoplastic tumor microenvironment. Enrichment scores are normalized from 0 (low enrichment) to 1.00 (maximum enrichment). Lower panels provide molecular descriptors for each functional category.

To define functional heterogeneity within the mesothelioma stroma, we characterized transcriptomic signatures across eight CAF subtypes (Figure 6A). Gene mapping analysis identified distinct subtype-specific programs associated with extracellular matrix remodeling, TGF-β signaling, immune regulation, angiogenesis, and metabolic adaptation. MatrixCAFs were enriched for structural ECM genes, whereas apCAF preferentially expressed immune regulatory programs. Functional enrichment analysis demonstrated strong subtype specialization, with high normalized enrichment scores observed across dominant biological programs (Figure 6B). VascularCAFs were preferentially associated with angiogenic pathways, whereas meCAFs exhibited enrichment for metabolic reprogramming signatures. Partial overlap among mCAF, TGF-βCAF, and pleuralCAF programs suggested coordinated contributions to extracellular matrix remodeling and desmoplastic niche formation within the mesothelioma tumor microenvironment.

### 3.7. Fibroblast–Mesothelioma Crosstalk Organizes EMT into Six Coordinated Molecular Modules

**Figure 7:**
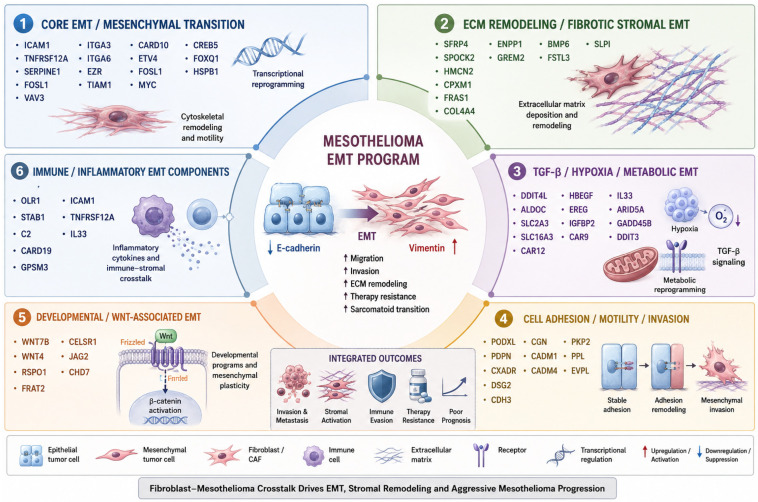
Fibroblast–mesothelioma crosstalk orchestrates EMT programs through six interconnected molecular modules. Schematic overview of the mesothelioma EMT program induced by fibroblast-derived cues. Six functional modules integrate transcriptional reprogramming, stromal remodeling, metabolic adaptation, developmental signaling, cell adhesion changes, and inflammatory crosstalk to promote mesenchymal transition, invasiveness, and therapy resistance in mesothelioma. (1) *Core EMT/mesenchymal transition.* EMT transcription factors, signaling mediators and cytoskeletal regulators that drive mesenchymal reprogramming, motility and invasion (*ICAM1*, *TNFRSF12A*, *SERPINE1*, *FOSL1*, *MYC*, *ITGA3*, *ITGA6*, *EZR*, *TIAM1*, *VAV3*, *CARD10*, *ETV4*, *CREB5*, *FOXQ1*, and *HSPB1*). (2) *ECM remodeling/fibrotic stromal EMT.* Extracellular matrix (ECM)-associated genes and fibroblast-enriched effectors that mediate matrix deposition, remodeling and desmoplastic responses (*SFRP4*, *SPOCK2*, *HMCN2*, *CPXM1*, *FRAS1*, *COL4A4*, *ENPP1*, *GREM2*, *BMP6*, *FSTL3*, and *SLPI*). (3) *TGF-β/hypoxia/metabolic EMT.* Genes linked to TGF-β signaling, hypoxic stress, and metabolic adaptation that promote EMT and support survival in a hostile microenvironment (*DDIT4L*, *ALDOC*, *SLC2A3*, *SLC16A3*, *CAR9*, *CAR12*, *HBEGF*, *EREG*, *IGFBP2*, *IL33*, *ARID5A*, *GADD45B*, and *DDIT3*). (4) *Cell adhesion/motility/invasion.* Molecules that regulate cell-cell and cell-matrix adhesion, polarity, and cytoskeletal dynamics to facilitate migratory and invasive behavior (*PODXL*, *PDPN*, *CXADR*, *DSG2*, *CDH3*, *CGN*, *CADM1*, *CADM4*, *PKP2*, *PPL*, and *EVPL*). (5) *Developmental/WNT-associated EMT.* Developmental pathways and WNT-Notch signaling effectors that sustain mesenchymal plasticity and stem-like programs (*WNT7B*, *WNT4*, *RSPO1*, *FRAT2*, *CELSR1*, *JAG2,* and *CHD7*). (6) *Immune/inflammatory EMT components.* Inflammatory mediators and immune-associated molecules that reinforce EMT through cytokine/NF-κB signaling and tumor–stromal crosstalk (*OLR1*, *STAB1*, *C2*, *CARD19*, *GPSM3*, *ICAM1*, *TNFRSF12A,* and *IL33*). At the center, fibroblast–mesothelioma interactions promote loss of epithelial characteristics (E-cadherin) and gain of mesenchymal features (vimentin), resulting in enhanced migration, invasion, ECM remodeling, therapy resistance, and sarcomatoid transition, as indicated by the upward arrows. The integrated outcome of these programs includes increased invasiveness and metastatic potential, stromal activation, immune evasion, treatment resistance, and poor clinical prognosis.

Fibroblast-derived cues induce a broad EMT program in mesothelioma cells that is structured into six interconnected functional modules (Figure 7). At the core is a canonical EMT module comprising transcriptional regulators and cytoskeletal effectors (*FOSL1*, *MYC*, *ETV4*, *CREB5*, *FOXQ1*, *EZR*, *TIAM1*, *VAV3*) that directly enforce mesenchymal identity, motility and invasion. This core program is reinforced by an ECM remodeling and fibrotic module, marked by induction of matrix-associated and stromal genes (*SFRP4*, *SPOCK2*, *COL4A4*, *FRAS1*, *CPXM1*, *GREM2*), consistent with desmoplastic remodeling of the tumor microenvironment.

A third module links TGF-β signaling, hypoxia and metabolic adaptation to EMT, coupling stress-response and metabolic genes (*SLC2A3*, *SLC16A3*, *CA9*, *DDIT4L*, *ALDOC*) with growth factor and cytokine mediators (*HBEGF*, *EREG*, *IGFBP2*, *IL33*). In parallel, extensive remodeling of cell–cell and cell-matrix adhesion (*PODXL*, *PDPN*, *CDH3*, *DSG2*, *CADM1/4*) supports migratory and invasive behavior. Fibroblast exposure also activates a developmental/WNT-associated module (*WNT7B*, *WNT4*, *RSPO1*, *FRAT2*, *JAG2*, *CHD7*), suggesting engagement of embryonic signaling pathways that stabilize mesenchymal plasticity. Finally, an immune and inflammatory module (*OLR1*, *STAB1*, *C2*, *ICAM1*, *TNFRSF12A*, *IL33*) integrates cytokine and NF-κBassociated signaling into the EMT network.

In summary, these six modules converge on loss of epithelial features, gain of mesenchymal traits, and enhanced migration, invasion, ECM remodeling and therapy resistance, providing a mechanistic framework linking fibroblast–tumor crosstalk to aggressive, sarcomatoid-like mesothelioma states.

## 4. Discussion

Our longitudinal profiling of mesothelioma progression reveals that TME is not a passive byproduct of growth but a proactively engineered sanctuary driven by a convergent stromal-EMT program. This study decouples the complexities of this progression into three functional pillars: EMT29, Stromal28 and Common14.

The EMT29 program represents the “invasive engine” of mesothelioma. Its overwhelming enrichment in EMT hallmarks and GO terms like integrin binding and stress fibers suggests that tumor cells acquire mesenchymal traits by physically anchoring to the stroma. This is mediated by a specific VCAM1-integrin αVβ5 axis, which senses matrix rigidity generated by LOX-mediated cross-linking. This creates a mechanical feed-forward loop: the stiffer the matrix, the more aggressive the EMT phenotype, which, in turn, leads to further matrix rigidification. While EMT29 drives invasion, the Stromal28 program, specifically enriched in pleuralCAFs, engineers the “fortified wall.” The unique prioritization of complement activation and IGF-binding (IGFBP5/7) suggests this program creates a protective corridor. By sequestering growth factors and triggering chronic inflammatory signaling, the Stromal28 niche likely serves as a physical and metabolic barrier that prevents T cell infiltration, providing a structural basis for the “immune desert” phenotype and subsequent resistance to checkpoint inhibitors. Associations with T-cell exclusion, immunotherapy resistance, sarcomatoid transition, and prognosis are inferred from prior studies and should be considered hypothesis-generating rather than definitive [19].

The Common14 program acts as the universal “scaffolding” required for tumor survival regardless of the specific niche. By maintaining protease balance (TIMP1/3) and metabolic coupling via gap junctions (GJA1/Cx43), this program ensures that both the tumor and its supporting matrixCAFs remain viable under the high-stress conditions of a rigidifying TME.

Distinct CAF subtypes organize the mesothelioma TME into spatially and functionally specialized niches, consistent with emerging single-cell and spatial transcriptomic studies demonstrating profound CAF heterogeneity [19]. MatrixCAFs and TGFβ-activated CAFs form the dominant structural backbone, driving extracellular matrix deposition, cross-linking, and mechanical stabilization-core features of tumor progression and therapy resistance [20]. These populations align with conserved myofibroblastic CAF programs that regulate ECM architecture and mechanotransduction across cancers [21]. In contrast, pleuralCAFs and vCAFs exhibit spatial specialization, linking site-specific fibrosis and angiogenic remodeling, reflecting the spatial compartmentalization of CAF functions revealed by modern multi-omics approaches [19]. Inflammatory and metabolic CAF subsets further diversify this ecosystem: iCAFs modulate immune signaling and complement activity, while meCAFs support metabolic adaptation and redox balance, both contributing to immunosuppressive and stress-adapted niches [19]. Notably, apCAFs remain transcriptionally distinct, consistent with reports that antigen-presenting CAFs represent a specialized, immune-interacting subset rather than core structural drivers [22]. Together, these findings support a hierarchical and cooperative CAF ecosystem underlying mesothelioma progression. CAF functional specializations are inferred from transcriptional profiles and remain associative. Definitive validation of these roles will require lineage-tracing and functional perturbation studies.

The mesothelioma TME can be conceptualized as three interconnected mechanoadaptive modules, EMT29, Stromal28, and Common14, that collectively integrate adhesion signaling, matrix remodeling, and stromal specialization into a unified genotype-to-niche framework [20,21]. EMT29 represents the primary invasive axis, coupling adhesion receptor-ligand networks with mechanotransduction. Within this module, VCAM1-α4β1 signaling sustains endothelial-pericyte interactions in inflammatory, hypoxic niches enriched for TNFα and IFNγ, while integrin-mediated sensing of ECM stiffening (ITGAV/ITGB5-SDC4) amplifies LOX and PLOD2-driven collagen remodeling, converging on YAP/TAZ-dependent invasion [20,23]. Stromal28 complements this by enforcing spatial niche specialization and immune modulation, with IGFBP5/7-mediated growth factor sequestration and mesothelial markers (MSLN, UPK family) defining fibrotic and immune-excluded territories [24]. These dynamic programs are stabilized by Common14, a conserved structural module that supports metabolic coupling via GJA1 and maintains ECM homeostasis through balanced MMP14 and TIMP1/3 activity [22]. Together, these modules establish a resilient, adaptive TME that promotes stromal protection and therapeutic resistance. These modules represent computationally defined signatures whose biological relevance and robustness across independent datasets require further validation.

These findings identify CAFs as central regulators of EMT-associated mesothelioma progression. In particular, matrixCAFs and TGFβ-CAFs emerged as dominant stromal populations linked to ECM remodeling, mechanotransduction, mesenchymal transition, invasive behavior, and immunoregulatory signaling. The substantial overlap between stromal and EMT-associated transcriptional programs further suggests that EMT in mesothelioma represents a dynamic tumor–stroma co-evolutionary process, rather than a purely tumor cell-intrinsic phenomenon.

Our co-culture experiments further demonstrate that fibroblast abundance is a major determinant of mesothelioma cell state and transcriptional plasticity. Single-cell RNA sequencing across varying tumor:fibroblast ratios revealed marked shifts in cellular composition and transcriptional programs, indicating that stromal density dynamically reshapes tumor behavior through direct tumor–stroma interactions. Fibroblast-rich conditions preferentially induced gene programs associated with extracellular matrix remodeling, inflammatory signaling, stress adaptation, and mesenchymal transition, consistent with activation of EMT-associated pathways. These findings are consistent with previous studies implicating EMT-associated transcriptional programs and stromal remodeling in aggressive mesothelioma progression [10,25].

Consistent with these observations, functional characterization of CAF populations revealed extensive stromal heterogeneity, with distinct CAF subtypes enriched for specialized tumor-supportive programs. MatrixCAFs were strongly associated with extracellular matrix remodeling, vascular CAFs with angiogenic signaling, and metabolic CAFs with metabolic adaptation, whereas TGFβ-CAFs and pleuralCAFs showed overlapping enrichment in ECM-related pathways linked to desmoplastic remodeling and mesenchymal progression. These data support a cooperative stromal framework in which functionally distinct CAF populations collectively shape the mesothelioma microenvironment through complementary but interconnected programs [10,25].

At the transcriptional level, the coordinated enrichment of EMT- and stromal-associated genes, including SERPINE1, PDPN, TNFRSF12A, FOSL1, HMGA1, IGFBP2, and SFRP4, further supports a model in which mesenchymal transition in mesothelioma is closely coupled to CAF activation, extracellular matrix remodeling, and mechanotransductive signaling [26,27,28]. In particular, PDPN and TNFRSF12A have previously been linked to invasive mesothelioma phenotypes, tumor motility, and poor clinical outcome [29,30]. Together, these findings suggest that matrix-associated and TGFβ-responsive CAF populations act as central regulators of tumor plasticity, invasion, and progression toward an aggressive sarcomatoid-like phenotype through reciprocal tumor–stroma crosstalk.

Our data suggest that CAF composition evolves over time, with early inflammatory-like CAFs gradually replaced by matrix-remodeling and myofibroblastic CAFs, consistent with established CAF heterogeneity. ECM-producing and TGFβ-associated CAFs become enriched at later stages, coinciding with activation of strong EMT programs [31].

These shifts align with distinct EMT states: inflammatory CAFs associate with partial EMT and stemness, whereas matrix-producing CAFs link to full EMT, desmoplasia, and invasion. Together, this indicates that both CAF identity and abundance shape EMT progression in mesothelioma [32]. Taken together, our findings define a unified mechanoadaptive framework in which mesothelioma progression is driven by the early and persistent coupling of stromal reprogramming, ECM remodeling, and EMT-associated plasticity [19,33]. Rather than emerging as late events, these processes are established at tumor initiation and maintained throughout disease evolution [20]. This convergence resolves into three coordinated programs (EMT29, Stromal28, and Common14) that, respectively, govern mechanotransduction and invasion, niche specialization and immune exclusion, and structural-metabolic stability [21]. At the cellular level, a hierarchically organized CAF ecosystem executes these programs, while at the tissue level, angiogenic and stiffness-driven niches reinforce a self-sustaining feedback loop linking genotype to microenvironmental adaptation [22,28,33].

Looking forward, targeting this integrated network will require strategies that disrupt both mechanical signaling and stromal support, including inhibition of ECM cross-linking, integrin/YAP pathways and CAF plasticity [19,20]. Integration of spatial multi-omics with functional perturbation studies will be critical to refine these vulnerabilities and enable translation into effective therapies [21,23].

Our study is primarily based on transcriptomic profiling, computational analyses, and co-culture experiments, which identify associations between CAF-associated programs and EMT-related transcriptional states rather than establish direct causality. Therefore, our findings should be interpreted as evidence of coordinated stromal and tumor-cell remodeling during mesothelioma progression. While the observed CAF–EMT associations are consistent with biological processes previously linked to tumor invasiveness, matrix remodeling, therapeutic resistance, sarcomatoid features, and adverse clinical outcomes, the present data do not directly demonstrate that these programs drive such phenotypes. Consequently, therapy resistance, poor prognosis, and sarcomatoid transition should be interpreted cautiously. Future studies incorporating functional perturbation approaches, invasion and drug-response assays, spatial validation, and clinical cohort analyses will be necessary to determine the causal contribution of specific CAF subtypes and EMT-associated programs to mesothelioma progression and treatment response.

## 5. Conclusions

Our longitudinal investigation into the murine mesothelioma microenvironment reveals a sophisticated stromal–EMT program that dictates disease progression. By integrating single-cell RNA sequencing with temporal transcriptomic profiling, we identified eight distinct CAF subtypes, including mCAF, vCAF, and apCAF populations, each characterized by unique molecular fingerprints and specialized functional identities. These CAF subsets do not operate in isolation; rather, they engage in a highly coordinated crosstalk with mesothelioma cells through six interconnected molecular modules: core mesenchymal reprogramming, fibrotic ECM remodeling, metabolic adaptation, developmental signaling (WNT/Notch), altered cell adhesion, and inflammatory signaling. This synergistic niche engineering facilitates the loss of epithelial identity and the gain of mesenchymal features, ultimately driving the desmoplastic transition, enhanced invasiveness, and therapy resistance. Our findings establish that the convergence of specialized CAF activities into a unified stromal–EMT axis is a fundamental determinant of the aggressive sarcomatoid transition and poor clinical prognosis in mesothelioma.

## Figures and Tables

**Table 1:** CAF subtype mapping of representative genes from each gene set in mesothelioma.

CAF Subtype	Genes from EMT29	Genes from Stromal28	Genes from Common14
myCAF (myofibroblastic CAF)	ACTA2, CALD1, TPM1, TPM2, CDH11	AEBP1, COL6A1, EFEMP1, OGN, SPON2	GJA1, SPARC, TGM2, FSTL1
matrixCAF (ECM-producing CAF)	COL3A1, COL5A2, LOX, PLOD2, SERPINH1, FBN1, FBLN5, MATN2, LAMC1, BMP1	COL6A1, EFEMP1, OGN, SPON2, LAMB1, CCDC80	BGN, DCN, FBLN1, FBLN2, LOXL1, PCOLCE, SPARC, MMP14, TIMP1, TIMP3
iCAF (inflammatory CAF)	VCAM1, IGFBP4, TNFRSF12A	C1S, C3, SERPING1, IGFBP5, IGFBP6, IGFBP7, CLU	TIMP1, FSTL1
TGFβ-CAF (TGFβ activated CAF)	ACTA2, COL3A1, COL5A2, LOX, PLOD2, SERPINH1, CDH11, TPM1, TPM2	AEBP1, COL6A1, EFEMP1, OGN, SPON2, IGFBP5	BGN, DCN, SPARC, TGM2, LOXL1, PCOLCE
vCAF (vascular CAF)	VEGFA, ITGAV, ITGB5	TM4SF1, SPON2, IGFBP7	SPARC, MMP14
pleuralCAF (mesothelial-derived CAF)	WNT5A, APLP1, DST, FERMT2, SDC4	MSLN, UPK1B, UPK3B, KRT19, CLDN15, GPM6A	GAS1, GJA1
meCAF (metabolic CAF)	IGFBP4, HTRA1	RBP1, GPX8, SMPD3, CPE	GAS1
apCAF (antigen-presenting CAF)	(none strongly represented)	(none clearly present)	(none clearly represented)

## Data Availability

All data generated or analyzed during this study are included in this published article.
